# Ethics in the crossfire: politicised patient care, cultural authority, and nurses’ constrained moral agency

**DOI:** 10.1186/s12912-026-05199-2

**Published:** 2026-08-10

**Authors:** Animesh Ghimire

**Affiliations:** 1 School of Nursing and School of Public Health, Chitwan Medical College, Bharatpur-5, Kailashnagar, Chitwan Nepal; 2 Sustainable Environment Foundation Nepal, Baneshwor- 31, Kathmandu, Nepal

**Keywords:** Nepal, Registered nurses, Patient care, Moral distress, Moral agency, Nursing ethics, Health policy, Reflexive thematic analysis

## Abstract

**Background:**

Socio-political polarisation increasingly shapes health decisions and clinical encounters, yet little is known about how nurses in low- and middle-income countries navigate conflicts among formal patient rights, family authority, cultural obligations, and politicised beliefs. Nepal provides a particularly important setting because rights-oriented legal reforms coexist with collectivist traditions, institutional resource constraints, and persistent social hierarchies. This study explored how registered nurses in Nepal interpret and manage ethically complex encounters in which patient care is influenced by political, legal, cultural, religious, or familial pressures.

**Methods:**

A qualitative study was conducted using in-depth, semi-structured interviews with 14 female registered nurses. Data were analysed using reflexive thematic analysis within an interpretivist and constructionist orientation; reporting was informed by the Reflexive Thematic Analysis Reporting Guidelines. The analysis combined semantic and latent engagement with the data, using moral distress and constrained moral agency as the principal ethical lenses.

**Results:**

Five interconnected themes were constructed: navigating legislative shifts in clinical practice; balancing personal values with nursing obligations; cultural priorities versus evidence-based care; systemic resource gaps and educational shortfalls—a widening chasm between policy and practice; and confronting hierarchies in nurse–patient interactions. Moral distress arose when nurses could discern a patient-centred or professionally responsible course of action but lacked clear policy guidance, institutional support, or sufficient authority to act. Family seniority, religious influence, political connections, and gendered assumptions could displace nurses’ clinical judgement. However, clear protocols, supportive senior clinicians, professional experience, and culturally negotiated care strengthened nurses’ moral agency and enabled ethical action.

**Conclusions:**

Politicised patient care in Nepal is not limited to overt partisan conflict; it is shaped by the interplay of law, culture, family power, gender, and organisational capacity. Legislative reform alone is insufficient unless healthcare institutions translate patient rights into workable procedures and protect nurses who advocate for ethically appropriate care. Context-specific ethics education, brief unit-based case discussions, clear escalation pathways, and gender-aware organisational support are practical measures that could reduce moral distress and strengthen ethical nursing practice in Nepal and comparable resource-constrained settings.

**Clinical trial number:**

Not applicable.

**Supplementary Information:**

The online version contains supplementary material available at https://doi.org/10.1186/s12912-026-05199-2.

## Introduction

Health care is no longer a strictly clinical environment; it is rapidly becoming a political space where issues of equity, individual autonomy, and social justice collide. Nurses, who deliver frontline care under increasingly complex circumstances, must balance professional ethics with external forces that strongly influence patients’ beliefs and behaviours. In Nepal, these dynamics are amplified by a dramatic constitutional shift from monarchy to federal democracy, paralleled by progressive legislation on reproductive health and LGBTQIA+ rights, yet offset by enduring collectivist values in the broader society. This study explores how registered nurses (RNs) in Nepal perceive and navigate ethical dilemmas that arise when patients’ socio-political views intersect with clinical decision-making—a phenomenon conceptualised here as the “politicised patient.”

### Background

Over the past decade, political polarisation has seeped into diverse domains of public life, including health care. While ideological debates once seemed distant from the bedside, they now increasingly shape treatment choices, patient expectations, and even institutional policies [1]. The International Council of Nurses (ICN) argues that nurses must reconcile the growing tension between legislation anchored in human rights frameworks and steadfast cultural norms [2]. This tension is particularly salient in nations undergoing rapid social transformations, where contradictions between new legal standards and traditional communal values can generate moral conflict.

Nepal exemplifies these tensions. In 2015, the country ratified a constitution that embraced federal governance and codified progressive notions of inclusivity [3]. Subsequently, Nepal became the first South Asian country to legalise same-sex marriage [4] and the second to legalise abortion [5], signalling a formal commitment to expanding personal freedoms and social justice. Nevertheless, multiple scholars note that widespread adherence to collectivist norms, familial authority, and hierarchical social roles remains prominent in much of Nepali society [4, 5]. For nurses, this confluence of progressive law-making and deeply rooted communal ideologies engenders recurrent ethical quandaries. These dilemmas become especially poignant when extended families, community elders, or religious figures openly challenge patients’ choices—even if said choices are fully sanctioned by new federal statutes.

### The “politicised patient” and nursing’s frontline role

The concept of the “politicised patient” emerges from the observation that patients’ health behaviours and acceptance or refusal of medical interventions can be driven as much by political ideology, legal shifts, or social activism as by strictly medical factors [6, 7]. In everyday clinical practice, this might appear when a patient refuses blood transfusions on religious grounds [8]; when families question vaccine efficacy on ideological grounds [9]; or when an individual’s end-of-life directives are shaped by politically charged debates about patient autonomy [10]. Such attitudes inevitably draw nurses into unfamiliar territory, requiring them to mediate conflicts, provide balanced health information, and uphold ethical care standards in ways that may depart from conventional clinical scripts.

In Nepal’s case, registered nurses hold a particularly complex role. They are expected to serve as both patient advocates and cultural negotiators, ensuring that individuals receive care informed by scientific evidence and aligned with international ethical principles, all while respecting local beliefs [11, 12]. RNs may, for instance, find themselves advising a patient who wishes to terminate a pregnancy in a setting where legal frameworks permit the procedure but community attitudes remain hostile. Alternatively, they might support the health needs of a same-sex couple, some of whose family members regard their relationship as culturally alien. In each scenario, the nurse’s role transcends routine bedside tasks and balances competing claims to morality, legality, and cultural solidarity.

### Nepal’s emerging federalism and its impact on nursing practice

Nepal’s evolution from a monarchy to a federal democratic republic aimed to decentralise governance and promote equitable service delivery. Under federalism, the state was reorganised into multiple provinces and local municipalities, each tasked with local health policy creation, resource allocation, and infrastructure management [13]. In theory, this local empowerment should translate into patient-centred healthcare strategies, with municipalities tailoring services to their communities’ specific epidemiological and cultural needs. Indeed, some provinces have leveraged newfound autonomy to pilot context-driven health initiatives or forge innovative partnerships with non-governmental organisations [14].

However, federal restructuring also introduced unintended complications. Scholars report uneven resource distribution, coordination gaps among provincial and national agencies, and a continuing shortage of specialised healthcare workers [13, 15]. Hospitals in major urban centres, while relatively well-equipped, still grapple with infrastructural constraints and fluctuating funding streams, leaving nurses under-resourced and overburdened [16]. Within these systemic challenges, legal reforms around reproductive and LGBTQIA+ rights can appear revolutionary on paper, yet often lack robust implementation frameworks on the ground [17, 18]. RNs thus confront a layered reality: they uphold legal statutes favouring autonomy and inclusivity, but cannot ignore entrenched social hierarchies that shape patient behaviour and family dynamics [19].

### Challenges in nursing education and ethical preparation

While federal reforms aim to enhance healthcare equity and local autonomy, nursing education in Nepal has struggled to keep pace with the changing ethical landscape [20]. Historically, formal nursing curricula have grounded students in fundamental principles—beneficence, autonomy, justice, and non-maleficence—drawing heavily on global codes of ethics [21]. Yet these foundational courses overlook the complexities of politicised conflict in a newly federal context. Courses often do not specifically address how to navigate family-driven decision-making that conflicts with best-practice guidelines, nor do they provide explicit strategies for confronting overt socio-political influences on patient care [22, 23].

In practice, newly graduated and experienced nurses alike rely on ad hoc experiential learning to develop communication and conflict-resolution skills, gleaning insights from mentors or through trial and error at the bedside. Some hospitals in urban centres have initiated staff development workshops on critical issues such as end-of-life care and reproductive counselling, but these training sessions frequently remain disjointed or optional [24]. This gap is magnified by the moral distress nurses can experience when they must carry out legally permissible interventions that clash with their own moral values or those of a dominant community group [25]. Consequently, RNs in Nepal face ethical dilemmas that call for more nuanced pedagogical approaches, ongoing professional support, and policy-level interventions designed to uphold quality, culturally adaptive care in an era of political flux.

### Research aim

The central objective of this study is to explore how registered nurses interpret and manage ethical dilemmas arising from political, legal, and sociocultural pressures in patient care. Amid progressive legislation and enduring cultural traditions, RNs often find themselves at the forefront of conflicts between official policies and entrenched communal or familial expectations. I intend to illuminate both the overt and subtle processes by which nurses reconcile their professional mandates, personal value systems, and the socio-political forces that influence patients’ health decisions.

To advance this goal, I address the following research question: How do registered nurses in Nepal navigate and reconcile the tensions when patient rights and legal mandates intersect with persistent communal beliefs, family structures, and personal moral standpoints?

## Theoretical orientation and research aim

### The reflexive thematic analysis: a big Q qualitative approach

Braun and Clarke’s [26] reflexive thematic analysis (RTA) is anchored in the premise that knowledge is not passively discovered but actively constructed through interpretive engagement with research data. Drawing on a “Big Q” qualitative stance [27], RTA departs from post-positivist methods that seek to validate findings through measures such as inter-coder reliability or consensus coding. Instead, it acknowledges the researcher’s intrinsic subjectivity as a valuable resource for generating nuanced themes. This orientation is particularly well-suited to contexts where legal and sociocultural shifts intersect, since it permits an in-depth exploration of the subtle, often latent layers of meaning that shape people’s perspectives on healthcare [28].

In adopting RTA for this study, I position my work within a broadly interpretivist and constructionist framework [29]. Rather than treating language or behaviour as transparent reflections of objective reality, I regard them as manifestations of evolving cultural, political, and professional discourses. This approach allows me to identify how nurses make sense of, adapt to, or at times resist legislative reforms and community expectations. While some aspects of analysis may focus on direct, semantic content—such as explicit references to new laws—an equally important focus is on latent themes revealing underlying attitudes, values, or tensions. The overarching aim is to develop a deep understanding of how registered nurses negotiate moral and professional boundaries in a rapidly changing society.

### The author’s positioning and reflexive practice

My professional and personal immersion in Nepal’s healthcare field has spanned the era of constitutional transition to federal governance and the implementation of progressive laws around reproductive rights and same-sex marriage. Over time, I have observed first-hand how these legal advancements may, on one hand, expand healthcare access and patient autonomy, while simultaneously provoking resistance among communities deeply rooted in collectivist norms. My dual role as a practitioner and someone closely involved in health policy conversations gives me an insider and analyst vantage point. It also makes it crucial to remain transparent about how these experiences might steer my interpretations.

To nurture reflexive openness [30], I have employed multiple strategies throughout the research process. One practice has involved sustained journaling, where I recorded personal reactions, observations, and evolving interpretations. Another strategy entailed deliberate engagement with colleagues and community members to solicit alternative viewpoints, thereby challenging or expanding my initial perceptions. I further explored visual reflections—constructing concept maps or diagrams—to illustrate the interplay between my background and the data I encountered. Sharing interim findings at academic workshops allowed me to receive critical feedback that illuminated potential biases. Finally, the method of critical incident analysis served as an additional layer of self-scrutiny [31], prompting me to closely examine how specific, memorable events shaped my assumptions about the ethical conflicts nurses encounter.

By integrating these reflexive practices, I aim to ensure that my thematic interpretations are both contextually grounded and critically examined. Rather than seeking to erase my influence on the research, I strive to make it explicit, consistent with RTA’s principle of regarding researcher subjectivity not as a flaw to be eliminated but as a lens that can deepen insight into the complexities of politicised patient care [32].

## Methodology

### Settings, participant selection and characteristics

#### Study setting

This study was conducted at Chitwan Medical College and B.P. Koirala Memorial Cancer Hospital, both located in Bharatpur, Nepal. These facilities serve as tertiary-level teaching hospitals, providing extensive healthcare services and advanced nursing education in an urban setting in Bagmati Province. This province is recognised as Nepal’s most developed region [34], with advanced healthcare infrastructure and a diverse patient population. The decision to focus on nurses working in these settings was driven by the expectation that urban hospitals, which often mirror broader social and policy shifts, would offer rich, multifaceted accounts of the ethical dilemmas nurses face amid rapidly evolving socio-political circumstances. Their exposure to a wide array of clinical cases and interactions with patients from varied backgrounds was anticipated to yield interpretive insights conducive to reflexive thematic analysis (RTA).

#### Participant selection and information power

An interpretive framework [33] grounded in information power [34] underpinned the decision to recruit a smaller yet contextually rich group of participants. Rather than assembling a numerically extensive dataset, I sought nurses with sufficient professional diversity and direct experience of ethical tensions triggered by socio-political factors. This approach is consistent with RTA, which values depth and interpretive potential over sampling approaches focused on representativeness or large sample sizes [35]. By focusing on nurses with considerable first-hand knowledge of daily moral and clinical challenges, the study aims to capture the nuanced realities of providing care amid shifting legislative and cultural norms.

#### Recruitment and selection

Recruitment notices were disseminated between January 2025 and April 2025 through the hospitals’ nursing communication channels, staff email lists, and physical noticeboards. The notices contained a concise description of the study, the eligibility criteria, the voluntary nature of participation, and a QR code linking to a secure online self-registration form. Hospital managers and nursing supervisors were not involved in selecting participants or determining who could register.

A total of 19 nurses completed the self-registration form. Of these, 15 met the eligibility criteria, 4 did not, and 1 subsequently declined or withdrew; 14 registered nurses ultimately participated in an interview. Because the invitation was distributed through open communication channels, the total number of nurses who viewed the recruitment notice could not be determined.

Eligibility required participants to (a) have at least three years of experience as a registered nurse and (b) have personally encountered a clinical situation in which a patient’s or family member’s political, cultural, religious, or moral position conflicted with recommended care, an institutional protocol, or a health policy. Criterion (a) was confirmed through the professional information entered on the registration form. Criterion (b) was assessed through a screening question asking prospective participants to confirm and briefly describe such an encounter and was reconfirmed before the substantive interview commenced. This process established that participants possessed direct experiential knowledge relevant to the research question without requiring them to disclose identifiable patient information during recruitment.

Participants practised across acute care, critical care, mental health, oncology, palliative care, community nursing, surgical, medical, emergency, and operating-theatre settings. This clinical breadth was sought to extend the dataset’s interpretive range rather than to achieve demographic or statistical representativeness. No additional exclusion criteria were applied beyond failure to meet the eligibility requirements.

#### Reflexive considerations in recruitment

My position as both a healthcare practitioner and an individual with policy-related engagements necessitated deliberate reflexivity regarding the power relations that could shape recruitment. At the time of the study, I was not employed by either study hospital. I held no supervisory, managerial, educational assessment, or performance evaluation authority over any participant. None of the participants was directly accountable to me in a professional capacity. My insider positioning therefore arose primarily from my professional familiarity with Nepal’s healthcare and policy environment rather than from institutional authority over the participant group.

The self-registration process was used to reduce the possibility that nurses would feel personally invited, selected, or professionally obliged to participate. Recruitment information expressly stated that participation was voluntary, that choosing not to participate would have no employment consequences, and that hospital managers would not be informed about individual decisions to participate. This reflexive stance resonates with RTA’s principle that knowledge is co-created [36] and recognises that researcher and participant positions can shape both willingness to participate and what can be spoken within an interview.

#### Participant characteristics

All 14 participants identified as female, reflecting the prevailing gender dynamics in Nepal’s nursing landscape. This observation is supported by WHO observatory data, which indicate that Nepal’s nursing workforce is entirely female [37]. Their ages ranged from 26 to 47 years, and their professional experience spanned 3 to 17 years. Nine nurses held Bachelor of Science (BSc) degrees in Nursing, and five had completed Master’s degrees in Nursing. No additional demographic variables—such as religious affiliations, caste/ethnicity, or detailed family data—were solicited, aligning with both ethical caution and the study’s interpretive scope. Because RTA emphasises how participants construct and negotiate meaning within their professional contexts [27], only attributes directly relevant to the interplay of nurses’ moral decision-making were retained. Table 1 lists these core demographic details and each participant’s primary area of practice, providing insights into the varied clinical environments in which participants encountered ethically complex situations.

**Table 1:** Socio-demographic characteristics of participants

Participant	Age	Gender	Previous Education	Years of Nursing	Area of Practice
P1	26	Female	BSc Nursing	3	Intensive Care Unit
P2	29	Female	BSc Nursing	6	Emergency Department
P3	32	Female	BSc Nursing	9	Community Nursing
P4	35	Female	Master’s in Nursing	12	General Practice (GP)
P5	28	Female	BSc Nursing	4	Oncology Nursing
P6	47	Female	Master’s in Nursing	17	Oncology Nursing
P7	30	Female	BSc Nursing	7	Palliative Care
P8	26	Female	BSc Nursing	3	Operating Theatre
P9	44	Female	Master’s in Nursing	14	Operating Theatre
P10	31	Female	BSc Nursing	8	Community Nursing
P11	27	Female	BSc Nursing	4	Surgical Ward
P12	41	Female	Master’s in Nursing	15	Psychiatry Ward
P13	33	Female	BSc Nursing	10	Medical Ward
P14	38	Female	Master’s in Nursing	13	Emergency Department

#### Ethical considerations

The Nepal Health Research Council (NHRC-321/2024) and the Institutional Review Boards of Chitwan Medical College and B.P. Koirala Memorial Cancer Hospital granted ethical approval for this study. Written and verbal informed consent was obtained from each participant.

The consent process addressed both the relational power dynamics of workplace-based recruitment and the possibility that recounting ethically difficult encounters could evoke discomfort, anxiety, guilt, or concern about professional repercussions. Participants were informed that they could decline to answer any question without giving a reason and without any effect on their employment or professional relationships. No identifiable information about participation or interview content was disclosed to hospital managers, supervisors, or colleagues. At the conclusion of the interview, participants were offered an opportunity to debrief, clarify concerns, and request the removal of any statement they did not wish to retain.

### Dataset generation

Semi-structured interviews were adopted as the principal means of data generation. This approach is grounded in an interpretivist and constructionist paradigm [38], wherein participants’ own words and reflections are essential to understanding how moral, cultural, and policy-related factors intersect in clinical decision-making. Open-ended prompts provided sufficient flexibility for participants to introduce unanticipated experiences and to elaborate on the contextual and relational dimensions of ethically complex encounters.

The interview guide comprised 10 open-ended prompts addressing encounters in which patient or family beliefs conflicted with recommended care; the influence of political, cultural, or religious positions on health decisions; tensions between personal values and professional obligations; the effects of legal and institutional policies; organisational support; and participants’ recommendations for nursing education and policy. The guide functioned as a flexible conversational resource rather than as a standardised questionnaire: the wording and sequence of questions could be adapted to the participant’s account, and follow-up prompts were used to clarify meaning and explore clinically or ethically significant experiences. No formal pilot interview was conducted. The complete interview guide is provided as Supplementary File 1.

All interviews were conducted in person in library rooms at the respective institutions. Each interview lasted approximately 45–60 min. Participants reconfirmed their consent before recording commenced and were reminded that participation remained voluntary throughout the interview.

Prior to the interview, participants accessed a digital plain-language statement outlining the study’s scope and the researcher’s professional background. At the beginning of each interview, I reiterated my professional positioning and invited questions about the research process. Rapport-building conversations concerning shared clinical interests and professional experiences preceded the substantive questions. This approach reflected the reflexive ethos of RTA and the co-created nature of interview data [39]. Throughout the interviews, I used active listening, clarification, and exploratory prompts to support participants in developing, reconsidering, or expanding their accounts.

### Data analysis

#### Reflexive thematic analysis

Reflexive Thematic Analysis Reporting Guidelines (RTARG) were selected to accommodate the interpretive and contextualised nature of this study (see Supplementary File 3). Unlike post-positivist paradigms that rely on consensus-based coding or reliability metrics, RTARG endorses a “Big Q” view of qualitative inquiry, positioning the researcher’s subjectivity as an instrument for deriving meaning. By allowing for deep, context-specific interpretive work, RTARG helped me illuminate the interplay among newly enacted policies, nurses’ personal values, and varied patient demands—a triad essential to understanding everyday ethical dilemmas in Nepalese clinical practice.

#### Data preparation

With participants’ permission, all interviews were audio-recorded. I undertook and reviewed the transcription and translation. I am a native Nepali speaker and am fluent in English through my nursing education, academic role, clinical practice, and health policy experience in Australia. This linguistic and professional background supported attention to nursing terminology, policy language, culturally embedded expressions, and the frequent code-switching between Nepali and English that characterised the interviews.

Recordings were initially transcribed in the language or combination of languages used by each participant, preserving switches between Nepali and English where these contributed to meaning. Nepali passages selected for analysis or quotation were then translated into English. I reviewed each translated passage against both the original-language transcript and the audio recording on numerous occasions, paying particular attention to idiomatic expressions, culturally specific concepts, emotional emphasis, and clinical terminology. Where a direct English equivalent was unavailable, I selected the closest contextually appropriate formulation and documented the interpretive decision in translation notes. The original-language transcript remained available throughout coding and theme development.

The translations were not reviewed by an independent translator. Research indicates that in cross-language qualitative studies, translation is not a simple, neutral transfer of words; rather, it is interpretive and may alter or diminish culturally embedded meanings [40]. To maintain conceptual—rather than merely literal—equivalence, I aimed for transparency by utilising original-language records, conducting repeated audio checks, and documenting my translation decisions [41, 42]. Contemporary methodological literature similarly cautions that translation timing and inconsistent translation practices can affect the cultural and conceptual meanings carried into qualitative analysis [40]. These procedures remained consistent with RTA’s commitment to contextual and interpretive engagement with participant accounts [26].

#### Analytic process

Following RTARG guidelines [28], the analytic process began with thorough, iterative readings of each transcript. This initial immersion enabled a comprehensive understanding of participants’ accounts, which were recorded in a reflexive journal to maintain an ongoing record of the researcher’s evolving interpretations and emotional responses. Coding commenced by identifying “interpretive segments,” or data portions that resonated with the study’s primary concern. These interpretive segments were annotated and refined through subsequent transcript reviews.

Meaning units—succinct passages reflecting a single idea or event—were first highlighted, ensuring that the raw essence of each participant’s statement remained intact. These meaning units then informed more focused interpretive segments that captured the broader or deeper implications of the raw text. Each segment was assigned an initial label, selected to signal its implied or explicit ethical tension or socio-political interplay. Multiple pass-throughs of the transcripts enabled the merging or redefinition of these labels, providing a stable yet fluid foundation for further thematic generation. Extensive reflexive journaling highlighted how my insider knowledge, personal and moral perspective, and professional background may have shaped the recognition or de-prioritisation of certain segments.

In keeping with the RTA principle, I did not employ external validation measures, such as consensus coding or peer debriefing, for reliability. Instead, the emphasis on reflexive openness underscored a critical awareness of how the data and analysis co-evolve [26]. By affirming my accountability in shaping thematic insights, the analytic process intentionally foregrounded interpretive nuance over the pursuit of universal agreement or objectivity [28].

#### Constructing themes

Reflexive Thematic Analysis conceives themes as actively generated rather than discovered [28]. Following multiple rounds of interpretive coding, I assembled the coding labels into cohesive groupings that signified shared meanings or patterns across participants’ narratives. Each grouping was then evaluated against the data to determine how it captured the moral, socio-political, or interpersonal tensions highlighted by participants. An illustrative progression from selected participant extracts to the final themes is provided in Supplementary file 2. In line with RTA’s focus on interpretive generation, terms such as “categories” or “sub-themes” were deliberately avoided. Rather than suggesting that the dataset contained predefined structures waiting to be uncovered, each theme was constructed through iterative engagement with participants’ accounts, my reflexive awareness of personal and contextual influences, and the ethical realities nurses face in a politicised healthcare environment. This approach acknowledges that themes represent provisional and context-bound understandings.

### Methodological rigour and reflexive coherence

According to the RTARG guideline, methodological rigour hinges upon reflexive transparency rather than standardised reliability checks. As the sole author and primary analyst, I approached the interpretive process with deliberate self-awareness, keeping a structured journal to capture evolving impressions and emotional responses to participant narratives. Acknowledging my dual role as a healthcare practitioner and policy researcher, I strove to make any predispositions that might shape thematic constructions visible, thus affirming RTA’s emphasis on the researcher’s subjective lens as a source of interpretive depth [27]. These reflexive strategies supplanted external validation measures, ensuring that themes emerged from ongoing dialogue between the dataset and my contextual understanding of Nepal’s socio-political landscape. Furthermore, I recognised the situatedness of this qualitative work: single-round interviews in an urban hospital context inevitably limit the scope of participant perspectives. Rather than construing these factors as gaps in representativeness, I accepted them as consistent with a “Big Q” viewpoint [26], whereby interpretive depth and contextual fidelity are valued over broad generalisation. This stance supports a careful examination of how registered nurses, operating within an evolving legislative framework, perceive, articulate, and negotiate ethically charged care scenarios—a focus that remains central to RTA’s commitment to interpretive richness.

## Results and discussion

**Fig. 1:**
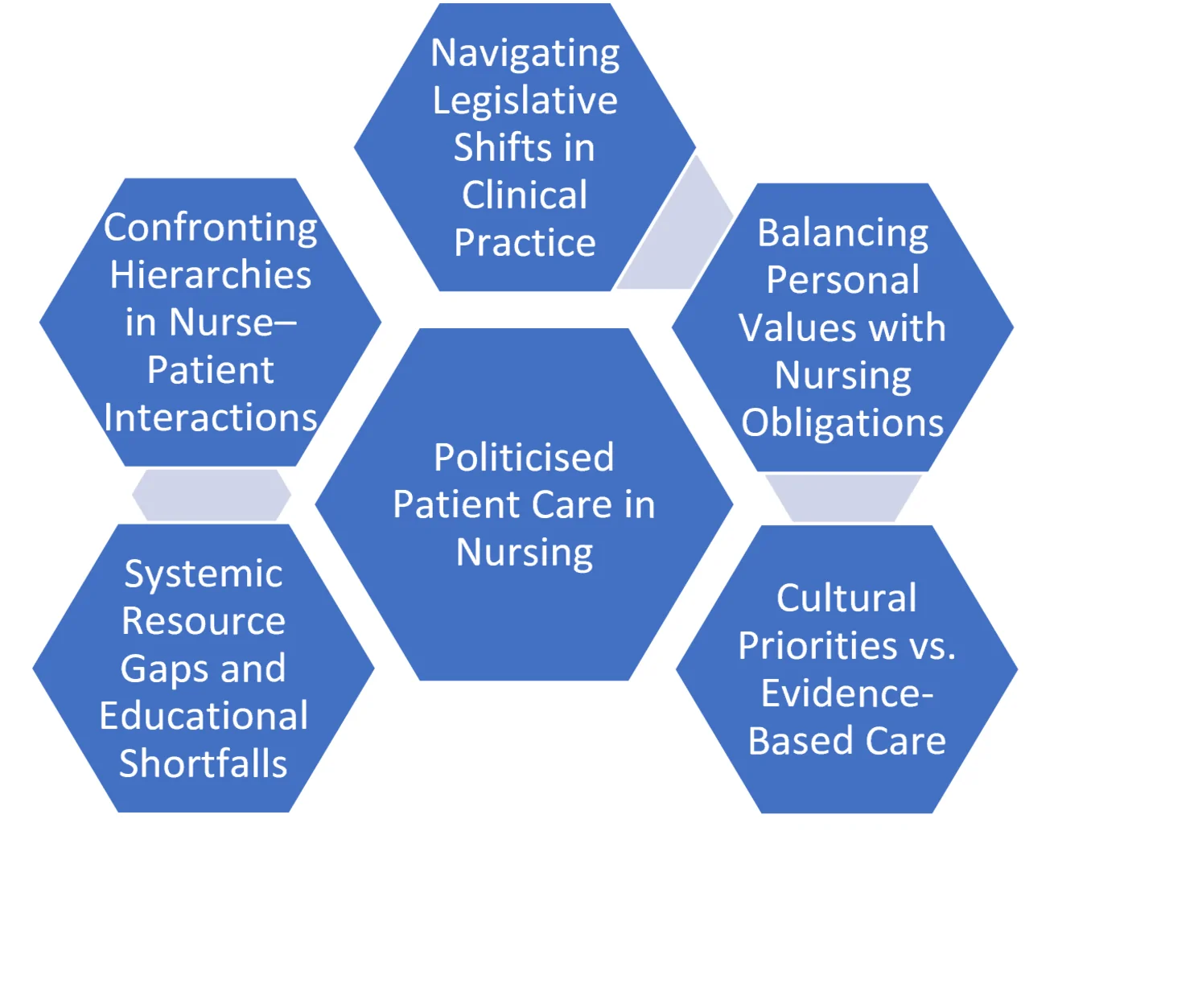
Overview of the five shared-meaning themes

Five shared-meaning themes were constructed through sustained reflexive engagement with participants’ accounts: (a) navigating legislative shifts in clinical practice; (b) balancing personal values with nursing obligations; (c) cultural priorities versus evidence-based care; (d) systemic resource gaps and educational shortfalls—a widening chasm between policy and practice; and (e) confronting hierarchies in nurse–patient interactions. Figure 1 presents the resulting thematic architecture. The themes are analytically distinct but interdependent. Across them, legislative ambiguity, personal conviction, communal authority, institutional insufficiency, and social hierarchy combine in different ways to expand or constrain nurses’ capacity to act on their ethical judgements.

Moral distress and constrained moral agency provide the principal ethical lens through which the five themes are interpreted. Moral distress is not used here as a synonym for discomfort, disagreement, or emotional difficulty. Rather, it refers to situations in which nurses formed a moral judgement about what patient-centred or professionally responsible action required but experienced personal, relational, organisational, or policy-level constraints on acting accordingly. When participants described uncertainty or value disagreement without impeding action, these experiences are interpreted as moral conflict or moral uncertainty rather than moral distress. This distinction avoids overstretching the concept of moral distress while recognising its relational and structural dimensions. Recent syntheses similarly locate nurses’ moral distress at the intersection of moral judgement, constrained agency, patient and family relationships, team dynamics, and institutional conditions [43–45].

### Theme 1: Navigating legislative shifts in clinical practice

This theme captures a central paradox: progressive legislation expanded formal recognition of patient rights, yet nurses’ practical capacity to enact those rights often remained uncertain. Legislative reform did not enter clinical practice as a clear and self-executing instruction. It arrived through incomplete institutional translation, divergent managerial interpretations, and encounters with families whose moral authority did not necessarily correspond with the individual-centred assumptions of formal policy. Nurses therefore occupied a liminal ethical space in which clinical judgement, policy directives, uncertainty, and value-laden expectations converge [46]. The shared meaning organising this theme was not just that “laws had changed,” but that nurses were made responsible for mediating reforms whose practical and relational consequences remained unresolved.

One nurse described the tension created when the patient’s expressed preference diverged from the family’s demand for continued intervention:

The law now says we must respect the patient’s wishes if they refuse certain interventions, but her entire extended family came, insisting we continue. I found myself in a bind: I respected her choice, but every day her relatives begged me not to let her stop treatment. It feels like carrying two flags—one for the hospital protocols and another for the family’s moral claims. (P1, ICU nurse)

The image of “carrying two flags” conveyed more than role conflict. It positioned the nurse as answerable to two moral constituencies whose expectations could not be simultaneously fulfilled. Their knowledge of the patient’s right to refuse treatment provided a moral judgement, but the extended family’s emotional pressure constrained the relational space in which they could act. This is consistent with moral distress understood as constrained moral agency rather than as the mere presence of competing values. Bourdieu’s account of social authority [47] helps explain why formal professional knowledge did not automatically prevail: the nurse was required to negotiate the symbolic power of family obligation alongside the normative power of policy.

Policy uncertainty was intensified when institutions failed to translate legal or professional guidance into consistent local procedures. A palliative care nurse explained:

Last year, we were told that palliative care decisions are more in the hands of the patient, per the new guidelines. But my superiors seemed uncertain about how far to go—should we press for a second opinion, or just trust the patient’s advance directive? Two different managers gave me two different instructions. I spent more time clarifying these ‘laws’ than I did caring for the patient. I realised that even the simplest tasks, like scheduling a do-not-resuscitate discussion, can become political in the sense that we’re all testing how these new mandates actually work. (P7, Palliative Care)

The participant did not reject the policy’s ethical orientation. The distress arose because responsibility for operationalising the guidance was transferred to a nurse working amid contradictory managerial instructions. The account illustrates moral dissonance but, more specifically, shows how institutional uncertainty restricts moral agency [48]. Foucault’s concept of governmentality remains useful here as a contextual account of how policy operates through local practices, documentation, and managerial interpretation [49]. It is not deployed as a separate ethical framework. Although the policy formally centred patient autonomy, inconsistent institutional interpretations reproduced uncertainty about who had the authority to decide and who would bear responsibility for the consequences.

Reproductive health generated a similar gap between legal recognition and socially permissible action:

There was this patient who came with her mother, wanting an abortion. Under the amended act, she was allowed to decide for herself. But her family demanded a sign-off from the father’s side. The mother said, ‘Legally, we can do it, but in our household, we don’t act alone.’ So, I stood there, uncertain if it was safer to slow down the process or remind them that she had the legal right. The tension was palpable. If I tried to be strictly ‘by the law,’ I might alienate them. If I waited, I’d undermine the patient’s autonomy. It’s like the system is telling us, ‘You have power now,’ and yet I feel powerless. (P10, Community Nurse)

“You have power now” contrasted sharply with “I feel powerless.” The law authorised advocacy, but it did not remove the social consequences of challenging family authority. Kleinman’s “local moral worlds” help contextualise why the patient’s legal autonomy was interpreted in terms of obligations to the household and kin [50]. Contemporary analysis of abortion policy similarly demonstrates that formal rights require ethically workable bedside procedures if they are to shape care rather than remain abstract legislative commitments [51]. The nurse’s uncertainty also reflects Derrida’s concern with undecidability [52]; applying a general rule required a situated judgement in which every available action risked a morally significant loss.

A general practice nurse offered a contrasting account in which legislative reform strengthened nursing agency:

Sometimes the policy actually acts as a protective boundary rather than a burden. We had an unmarried woman present for a sensitive reproductive health consult, and her elder male relatives insisted on sitting in, which violates her legal right to privacy. In this instance, however, our clinic had recently implemented a definitive, step-by-step protocol for enforcing patient confidentiality under the new act. My clinical manager stepped in beside me, pointing directly to the institutional mandate. Because I wasn’t fighting that battle alone, I could respectfully ask the relatives to wait outside without absorbing all their frustration. It showed me that when the institution actually shoulders the weight of the law, we can do our jobs ethically and safely. (P4, General Practice)

This shows that moral distress was not an inevitable consequence of cultural difference or legislative change. When legal provisions were translated into accessible institutional guidance and supported by senior clinicians, policy could strengthen nurses’ moral agency. The contrast therefore locates the central problem in uneven implementation rather than in progressive legislation itself.

Taken together, these accounts show that legislative reform reorganised the moral terrain of nursing practice without necessarily supplying the institutional resources required to navigate it. Research on Nepalese nurse managers similarly demonstrates that policy expectations can leave nurses responsible for ethically consequential decisions without commensurate organisational capacity [53]. Differentiated nursing practice can expand professional agency when authority and accountability are made explicit [52], whereas unclear authority can leave nurses negotiating ethical dilemmas at the bedside with little practical protection [54]. Policy success consequently depends not only on legislative formulation but also on whether nurses can enact policy without coercing patients, abandoning families, or becoming the sole absorbers of institutional uncertainty [55, 56].

### Theme 2: Balancing personal values with nursing obligations

The central organising meaning of this theme was that professional neutrality did not require moral emptiness. Participants remained morally situated individuals whose religious, political, and cultural commitments entered the clinical encounter, even when they deliberately prevented those commitments from directing patient care. Their challenge was not to eliminate personal values but to distinguish between having a moral response and claiming the authority to impose that response on a patient. Moral psychology [57] helps explain the durability of intuitive moral reactions, while feminist ethics of care [58] draws attention to the relational and gendered moral labour involved in maintaining attentive care amid disagreement.

A community nurse described the emotional effort of maintaining professional obligations when family demands conflicted with her faith:

I felt torn when the patient’s family insisted on continuing an intervention that I personally opposed […]. We all have professional duties, but I couldn’t pretend my own faith had disappeared just because I put on my scrubs. The hospital is clear that we must not allow personal beliefs to compromise care, yet I found it emotionally draining to comply. At times, I questioned whether I was betraying my moral self or simply fulfilling the nurse’s role. I kept thinking, “Am I truly respecting both the patient and my faith, or am I just following the handbook? (P3, Community Nurse)

The account exposed a conflict between moral identities, but it does not automatically constitute moral distress. The nurse remained able to provide care consistent with professional standards. Their experience is better understood initially as moral conflict: professional action remained possible, although emotionally costly. It approached moral distress when institutional expectations left no legitimate space to acknowledge, examine, or process the conflict. Ethical bedside decision-making often requires nurses to act despite unresolved moral discomfort [54, 59] and Draper’s [60] conception of embodied nursing practice clarifies why professional identity cannot be detached from the practitioner’s moral biography.

One oncology nurse similarly observed, “*I have no issue with the policy in principle*,* but it does weigh on me when the family’s request challenges my personal sense of right and wrong*” (P6, Oncology nurse). The phrase “weigh on me” conveyed a sense of moral residue rather than a refusal to fulfil professional obligations. Carper’s [61] personal way of knowing is relevant because self-awareness allowed the nurse to recognise their reaction before it shaped care. Such awareness becomes ethically productive when it supports disciplined reflection rather than elevating the nurse’s values over the patient’s.

A more explicitly political encounter required an emergency nurse to recognise and interrupt her own judgement:

There was a young man who came in after a riot. He was associated with some political group whose values I deeply opposed. My job mandated neutrality, and logically, I knew I had to offer him the same compassionate care as anyone else. But a subtle bias kept cropping up, telling me he didn’t deserve preferential attention. That realisation was painful: was I, as a nurse, letting my political stance affect the care I delivered? Stepping back, I reminded myself that professional ethics serve everyone equally, yet my personal sentiments didn’t vanish just because the code of ethics said so. (P14, Emergency Department)

This account expands the politicised patient beyond disputes over legislation. The patient’s perceived political identity activated a moral judgement in the nurse, but the nurse’s reflexive recognition of that judgement enabled rather than obstructed moral agency. They re-centred the patient’s entitlement to equitable care. Morley and Sankary [44] caution that moral agency should not be conceptualised as the unrestricted enactment of nurses’ own values; ethically appropriate nursing agency remains oriented toward the patient’s values, rights, and welfare.

Institutional culture shaped whether these experiences remained manageable moral tensions or became sources of moral distress. Some nurses described access to colleagues or ethics-oriented discussions that legitimised reflective processing. Others encountered the managerial statement, “*there’s no room for your personal stance here*” (P5, Oncology Nurse). Although this directive protected patients from discriminatory care, it also risked treating nurses as morally interchangeable functionaries. Peter and Liaschenko’s [62] analysis of moral ambiguity and proximity explains why nurses who remain continuously present at the bedside may carry an emotional burden not resolved by procedural instruction. The ICN [63] requires nurses to preserve patient dignity and professional integrity; neither obligation is served by imposing nurses’ beliefs on patients, nor is it strengthened by denying that morally difficult care affects the practitioner.

The theme therefore concerns disciplined moral integration rather than compromise between equally authoritative preferences. Nurses’ personal beliefs persisted, but professional responsibility required them to place patient rights and welfare at the centre of their actions. Moral distress intensified when nurses had no legitimate means to process the resulting conflict, or when social and institutional constraints prevented them from acting consistently with their professional judgement.

### Theme 3: Cultural priorities versus evidence-based care

This theme was organised around the question of whose knowledge and authority became actionable at the bedside. Participants described encounters in which biomedical recommendations were interpreted through family obligation, modesty, spiritual belief, stigma, and financial feasibility. The analysis does not position culture as an irrational obstacle to science. Rather, it shows that evidence becomes clinically effective only when it can be negotiated within the moral relationships through which patients understand illness and authorise care. Moral distress arose when nurses recognised an avoidable clinical risk but lacked the relational or institutional authority to act on that judgement.

One participant described a family’s refusal of a clinically indicated examination:

My patient’s family refused to let her undergo a gynaecological exam, saying it would violate her modesty. I knew from the guidelines that she needed a thorough check, but her relatives insisted they had the final say as elders. They told me, “We handle women’s issues within the family. Let us do it our way.” I felt torn: clinically, ignoring potential complications was risky, but culturally, insisting on an exam could be perceived as disrespect. The family expected me to yield to their hierarchy. Caught in the middle, I tried explaining the potential dangers of missing an early diagnosis, but they looked at me like I was insulting their traditions. (P14, Emergency department)

The nurse’s distress did not stem simply from encountering a different understanding of modesty. It arose because the nurse judged that delayed assessment could cause harm while the family’s authority constrained her ability to secure informed, patient-centred decision-making. Transcultural nursing scholarship emphasises the need to understand cultural meanings rather than to apply biomedical standards without dialogue [64]. At the same time, culturally responsive practice should not require nurses to remain silent where clinically significant risk is present. Hierarchical family decision-making remains influential in Nepal [65], and South Asian bioethics has long questioned the uncritical transfer of individualistic autonomy models into relational settings [11, 66]. The nurse consequently acted as a cross-cultural translator, but translation did not guarantee that her clinical judgement would gain authority.

Mental health encounters revealed an additional intersection among spiritual explanation, social stigma, cost, and clinical risk:

In our setting, depression is often treated as something you can pray away or ignore if you’re strong enough. A patient came to me with acute depression and suicidal thoughts, but their mother insisted it was “just a phase” and argued that “we don’t do therapy, we do devotion” […]. I wanted to refer them to a psychiatrist, as per hospital policy, but the family declined, saying the cost was unnecessary. It was heart-breaking. I had to find a middle ground, maybe scheduling a confidential counselling session so they wouldn’t feel ashamed. You realise how powerless strict guidelines can be if the family thinks your approach is culturally alien. (P12, Psychiatry Ward)

The participant’s attempt to arrange discreet counselling reflected practical moral agency under constraint. The nurse did not dismiss devotion as meaningless, yet they could not treat spiritual coping as an adequate response to suicidal risk. Recent work on mental health and faith advocates cultivating common ground rather than an oppositional framing of religion and clinical care [67]. The account also demonstrates why the conflict cannot be explained by belief alone: treatment cost and fear of social exposure reinforced the family’s rejection. Psychiatric categories may themselves be culturally interpreted [68], but the nurse remained ethically accountable for foreseeable harm. Their “middle ground” was therefore neither complete policy compliance nor passive cultural accommodation; it was an improvised effort to preserve access to care.

Nevertheless, cultural and spiritual commitments also supported clinical care:

Families often bring blessed threads (dhaago) or amulets and insist the patient wear them during surgery to ward off complications. A strict biomedical approach would be to simply cut them off to maintain sterility. But we negotiate. In one orthopaedic case, the parents were distraught at the idea of removing their child’s protective thread. I explained the infection control protocols, and we agreed to securely tape the thread high up on the shoulder, well away from the sterile field. They felt their child was spiritually protected, and we maintained our clinical safety standards. Accommodating their ritual built immense trust for the post-operative recovery. (P8, Operating Theatre)

This contrasting account resists a deficit portrayal of Nepali culture. It demonstrates that culture served as a relational resource, enabling nurses and families to negotiate complementary practices without compromising safety. It also clarifies that the source of moral distress was not collectivism itself but situations in which moral, economic, or hierarchical constraints foreclosed clinically defensible options. The theme therefore reframes evidence-based care as a relational accomplishment. Nurses required clinical knowledge, cultural humility, communication skills, and sufficient authority to negotiate care without either romanticising tradition or treating biomedical knowledge as culturally neutral.

### Theme 4: Systemic resource gaps and educational shortfalls: a widening chasm between policy and practice

The shared meaning uniting this theme was that policy without implementation infrastructure transferred ethical responsibility downward to individual nurses. Participants were expected to protect autonomy, correct misinformation, mediate family conflict, and deliver culturally responsive evidence-based care, yet many lacked clear protocols, accessible ethics consultation, protected learning time, or structured escalation pathways. Under these circumstances, moral distress was not primarily a deficit of individual resilience. It was a predictable response to institutional conditions that demanded ethical action while restricting the resources and authority needed to perform it. This interpretation is consistent with health-governance scholarship showing that macro-level policy goals can stall when organisational learning and local implementation capacity remain underdeveloped [69, 70]. Within Nepal, workforce shortages and constrained clinical education compound the ethical consequences of this policy–practice divide [15].

A surgical nurse described the inadequacy of generic professionalism training when confronting patriarchal authority:

We do have seminars on ‘patient-centred care,’ but none ever touched issues like dealing with family members who refuse treatment on the basis of “women should not decide alone.” I vividly remember standing there, hearing this man proclaim that only men in the house could consent to any procedure, and I realised we never addressed such deep-rooted biases in our continuing education. Managers told me, “Just be professional,” but it’s not that simple when family beliefs override everything, including my clinical recommendations. I craved step-by-step approaches or conflict-resolution techniques that factor in cultural hierarchies, but all I got were generic guidelines to ‘keep calm.’ (P11, Surgical Ward).

“Keep calm” individualised a systemic problem. It instructed the nurse to regulate their behaviour without providing a procedure to protect the patient’s agency or to address discriminatory family conduct. Situated learning is relevant because nurses need opportunities to rehearse communication, escalation, and ethical reasoning within cases that resemble their clinical reality [71]. Without such preparation, the burden of translating general principles into action remained with the individual nurse at the moment of conflict.

An emergency nurse similarly distinguished the existence of policy from the availability of an actionable framework:

It’s not that the hospital policy is silent on respecting patient beliefs; it’s just that we have no real protocol for resolving deeper conflicts. If the family cites religious or political reasons […], the policy says we must ‘respect their view but prioritise patient welfare.’ That sounds wonderful on paper, but how do I handle it if the patient’s life is at risk? Do I stand by, or do I try to force care? Sometimes I watch colleagues trying to readjust what “respect” means on the fly, hoping we don’t violate our nursing license. (P14, Emergency Department)

The nurse was not asking for a script capable of resolving every moral conflict. They were asking for an institutionally recognised process that clarified consultation, documentation, escalation, and professional protection. International financing and education-reform literature similarly indicates that rights-oriented policy requires investment in workforce capability and implementation systems [72, 73]. Moral distress scholarship supports this interpretation: nurses report distress when they possess responsibility without autonomy, specialist knowledge, or organisational routes for raising concerns [43, 45].

A medical ward nurse described the disconnection between top-down policy language and local implementation:

We hear from the top—‘Implement patient-centred ethics, follow the national policy that respects choice’—but we have no specialised training to handle families who brand certain vaccines or treatments as “government conspiracies.” Also, because many of us come from the same local communities, pushing the official line can cause friction with our relatives or neighbours[…]. We have policies telling us to stand firm, but real empowerment demands that we have both the knowledge and institutional backing to do so. Right now, it feels like policy points in a direction, without helping us cross the cultural gap. (P13, Medical Ward)

The metaphor of policy “pointing in a direction” captured implementation without accompaniment. Nurses were positioned as policy agents, but organisational change is unlikely to be sustained when staff lack protected learning time, local leadership, and meaningful involvement in implementation. Unsupportive work environments also intensify stress and compromise the conditions required for ethical action.

Ultimately, these participants’ accounts reveal that bridging the gap between policy ideals and actual nursing practice in Nepal requires more than superficial references to “professionalism.” Confronted with politicised misinformation, culturally sanctioned patriarchal structures, or the unpredictability of low-resource environments, nurses consistently voiced the need for specialised, context-relevant training and institutional supports that engage with the moral texture of everyday clinical work. By illuminating these shortcomings, this theme underscores how systemic reforms—if embedded in local realities and comprehensive educational efforts—can catalyse more ethically coherent care for politicised patients in Nepal and comparable LMIC settings.

### Theme 5: Confronting hierarchies in nurse–patient interactions

The central meaning of this theme was that nursing authority remained socially conditional. A nurse’s clinical knowledge did not carry equal weight in every encounter; its legitimacy was mediated by age, gender, professional status, family seniority, religious standing, and political connections. These hierarchies were not external background factors. They entered clinical decisions directly, determining whose interpretation was heard, who could challenge a family decision, and whose account of patient welfare became actionable.

A community nurse described how an elder’s spiritual authority displaced her clinical recommendation:

Our facility tries to encourage direct nurse–patient communication, but one day I arrived to find a community elder telling the patient, “Do not listen to them if they propose modern tests for your condition. We’ve seen many healings if you follow this astrologer’s advice […].” Because this elder carried more moral weight than I did, the entire family followed his cues. I realised my official role and documented expertise meant little when faced with culturally recognised authority. It’s not like I can just say, “He’s wrong,” because that might be seen as insulting their beliefs. Although I had updated guidelines on her chronic condition, none of it had practical leverage in a situation where I was outranked socially. (P10, Community Nursing)

Hospital ethnography demonstrates that clinical settings remain embedded in broader social relations rather than operating as neutral technical spaces [74]. Bourdieu’s symbolic capital provides a useful sociological explanation for why the elder’s accumulated moral status outweighed the nurse’s credentialed expertise [75]. The ethical significance, however, lies in constrained moral agency: the nurse judged that further investigation was clinically warranted but anticipated that direct challenge could rupture trust and diminish her capacity to influence care.

Age and gender intersected with professional hierarchy. One operating-theatre nurse observed that “*if you’re young*,* they think you’re inexperienced*,* so they’ll push you to the side and say*,* ‘We want to talk to a senior male doctor who understands us’*” (P9, Operating Theatre). Because all participants in this study were women, this recurrent preference for an older male physician warrants explicit attention. The dataset cannot establish how male nurses would experience comparable encounters, but it demonstrates how the feminised status of nursing can interact with patriarchal assumptions to weaken women nurses’ authority. Recent research continues to document gender stereotyping in nurses’ professional experiences [76], while a systematic review found that gendered expectations, organisational structures, and perceptions of women’s subordinate position restrict nurses’ leadership and influence [77]. Family-centred decision-making can further intensify these tensions when collective authority overrides the individual patient’s preference [78].

Hierarchy also entered the clinical encounter through political connection:

If I escalate a disagreement to my department head, someone might mention that the patient’s brother-in-law is a local political figure. We risk potential fallout if they file a complaint about me being “disrespectful.” In some cases, the senior nurse says, “Try not to inflame them,” which is code for, “Don’t do anything that disrupts family hierarchy, even if it jeopardises the best practice.” It’s a constant struggle. We know what’s clinically needed, but political connections or social ranking can overrule it. (P2, Emergency Department)

The instruction not to “inflame” the family transformed organisational risk management into a constraint on nursing advocacy. Foucault’s account of dispersed power helps explain how political influence operated without a formal directive [79]: anticipated complaint and reputational risk were sufficient to narrow the range of actions considered safe. This is a particularly clear instance of moral distress. The nurse articulated a judgement about clinically appropriate care yet perceived that social power and limited organisational protection prevented assertive advocacy.

However, an alternative view from the participant demonstrates that seniority, specialist knowledge, and established professional relationships can sometimes alter these restrictive dynamics:

When you are a junior nurse, a local political leader or a senior male relative can silence you with a single look. But after seventeen years on the oncology ward, my experience carries its own weight. Recently, a family member tried to dictate his wife’s care plan […]. Because I have worked alongside our department head for over a decade, I knew I had unconditional institutional backing. I pulled the husband aside and spoke to him not just as a subordinate caregiver, but as a senior specialist who has guided hundreds of families through this exact transition. I firmly stated that our clinical duty was to his wife’s comfort, and we would not compromise it. He eventually agreed. But that level of advocacy is a privilege of my seniority. (P6, Oncology Nursing)

Such an account illustrates that hierarchy is neither uniform nor entirely impermeable. Experience and relational credibility can provide forms of professional capital that expand a nurse’s room for action. Recent research involving Nepali nurses also links healthcare hierarchy and the undervaluation of nursing expertise to professional identity and decisions about whether to remain in the national workforce [80]. Confronting hierarchy therefore involved more than improving nurses’ communication skills. It required recognition that clinical authority is gendered, relational, and politically situated. Strengthening ethical nursing practice in Nepal will depend on institutional arrangements that affirm nurses’ expertise, provide escalation pathways when influential families exert pressure, and enable nurses to advocate without carrying the personal risk of challenging entrenched authority alone [69, 70].

## Final considerations

This study shows that the ethical challenges associated with politicised patient care in Nepal do not arise solely from overt party-political beliefs. They are produced through the interaction of legal reform, family authority, religious and cultural commitments, institutional ambiguity, and unequal organisational support. Across the five themes, registered nurses were positioned as mediators of tensions that policy had not fully resolved. Moral distress intensified when nurses could recognise a patient-centred course of action but lacked the authority, procedural clarity, or institutional backing needed to pursue it. Conversely, where policies were translated into clear local procedures and supported by senior clinicians, nurses’ moral agency was strengthened. The central contribution is to move beyond individual resilience or interpersonal disagreement: the ethical consequences of socio-political change depend in part on whether healthcare organisations equip nurses to translate formal rights into workable clinical practice.

The all-female participant group is analytically important, reflecting the feminised structure of nursing in Nepal [37]. Their accounts were generated in contexts where nursing expertise may be subordinated to older men, senior physicians, family patriarchs, religious figures, or politically connected individuals. Families’ preference for senior male doctors, and the reduced credibility assigned to younger women nurses, indicate that constrained moral agency was also gendered [81]. Feminist ethics of care illuminate these inequalities by directing attention to care labour, voice, dependency, and unequal authority [58]; however, it is used as a contextual perspective rather than as a competing ethical framework. Moral distress and constrained moral agency remain the principal interpretive lens. The gender composition also delimits the knowledge produced: this study cannot establish whether male or gender-diverse nurses encounter these hierarchies similarly. Recent scholarship similarly shows that women nurses’ authority is shaped by gendered expectations, not clinical competence alone [77, 82].

The findings support practical measures that do not require extensive new infrastructure. Nursing leaders could introduce brief unit-based ethics discussions centred on anonymised cases involving family pressure, misinformation, reproductive rights, end-of-life decisions, or discriminatory conduct. These sessions should move beyond instructions to “remain professional” and allow nurses to rehearse respectful challenge, documentation, escalation, and culturally responsive communication. Hospitals could also produce concise policy-to-practice guides specifying who should be contacted, what should be recorded, and how nurses will be supported when patient rights or clinical safety are threatened. A named senior nurse or on-call ethics contact could offer rapid consultation, while short post-incident debriefs could provide space to process moral residue. These measures align with the International Council of Nurses’ emphasis on dignity and supportive ethical environments [63] and respond to organisational moral distress documented in Nepalese nursing [53].

Educational reform should be equally concrete. Continuing professional development and preregistration curricula should use locally grounded scenarios rather than decontextualised ethical principles alone. Simulation and case discussion could address situations in which autonomy is interpreted through family relationships, political or religious misinformation affects treatment decisions, or a nurse’s gender and age diminish her authority. The purpose should not be to train nurses to defeat culture through better argument, but to help them identify the patient’s preference, distinguish cultural accommodation from preventable harm, negotiate with influential relatives or community figures, and seek institutional support before conflict escalates. Public health communication developed with community leaders, women’s organisations, patient advocates, and local government could reduce the burden placed on individual nurses to correct distrust at the bedside.

Future research should follow from the conditions revealed here. Longitudinal studies could examine whether repeated exposure to policy change strengthens confidence or produces cumulative moral residue. Comparative inquiry across public, private, rural, and urban facilities could explore how staffing, leadership, ethics support, and local political relationships shape nurses’ capacity to act. The gendered findings warrant research involving male and gender-diverse nurses, not merely to repeat this study in another group, but to examine whether professional credibility and exposure to patriarchal authority vary by gender. Participatory research with patients, family decision-makers, religious leaders, nurse managers, and policymakers could identify where interpretations of autonomy, respectful care, and legitimate authority converge or remain irreconcilable.

The reflexive thematic analysis generated situated interpretations rather than statistically generalisable claims. My insider knowledge of Nepalese healthcare and policy enabled culturally informed interpretation while requiring sustained reflexive scrutiny. The urban tertiary hospital setting, single interviews, and an all-female participant group shaped what could be spoken and interpreted; these features define the study’s analytic reach rather than constituting simple deficiencies. Taken together, the findings show that legislative progress will remain ethically fragile when nurses are expected to absorb unresolved tensions among policy, culture, and institutional limitation. Bridging this divide requires policy clarity, gender-aware organisational protection, context-sensitive ethics education, and accessible routes for consultation. Such measures would not eliminate moral disagreement, but they could ensure that nurses are not left to carry its consequences alone.

## Supplementary Information

Below is the link to the electronic supplementary material.

Supplementary Material 1

Supplementary Material 2

Supplementary Material 3

## Data Availability

The data supporting this study’s findings are available on request from the corresponding author. However, the data is not publicly available due to privacy or ethical restrictions.
