# Supplementary material for: Ethics in the crossfire: politicised patient care, cultural authority, and nurses’ constrained moral agency
Source: BMC Nurs. 2026 Aug 10;25:1080. doi: 10.1186/s12912-026-05199-2 (PMC13741607; doi:10.1186/s12912-026-05199-2)
Supplement: Supplementary file 2 [file 12912_2026_5199_MOESM1_ESM.docx]

**Supplementary Table S2. Illustrative Progression From Meaning Units to Final Themes**

| **Meaning unit: brief participant extract** | **Initial interpretive segment** | **Initial coding label** | **Developing shared-meaning pattern** | **Final theme** |
| --- | --- | --- | --- | --- |
| “It feels like carrying two flags—one for the hospital protocols and another for the family’s moral claims.” **(P1, Intensive Care Unit)** | The nurse experiences simultaneous accountability to patient-rights policy and the family’s moral expectations. | Dual moral accountability | Enacting policy while constrained by familial authority | **Navigating Legislative Shifts in Clinical Practice** |
| “When the institution actually shoulders the weight of the law, we can do our jobs ethically and safely.” **(P4, General Practice)** | Clear organisational procedures and managerial support transform legislation into an actionable source of protection. | Institutionally supported policy enactment | Organisational implementation expands nursing moral agency | **Navigating Legislative Shifts in Clinical Practice** |
| “I couldn’t pretend my own faith had disappeared just because I put on my scrubs.” **(P3, Community Nursing)** | Personal faith remains morally and emotionally present despite the nurse’s commitment to professionally appropriate care. | Enduring moral identity | Integrating personal convictions without imposing them on patients | **Balancing Personal Values with Nursing Obligations** |
| “My personal sentiments didn’t vanish just because the code of ethics said so.” **(P14, Emergency Department)** | The nurse recognises a politically influenced judgement but deliberately re-centres the patient’s entitlement to equitable care. | Reflexive interruption of bias | Professional obligations discipline rather than erase personal moral responses | **Balancing Personal Values with Nursing Obligations** |
| “You realise how powerless strict guidelines can be if the family thinks your approach is culturally alien.” **(P12, Psychiatry Ward)** | Clinical guidance loses practical force when the family interprets mental health care as culturally or spiritually incompatible. | Cultural alienation of clinical guidance | Relational and cultural barriers constrain evidence-based intervention | **Cultural Priorities versus Evidence-Based Care** |
| “Accommodating their ritual built immense trust for the post-operative recovery.” **(P8, Operating Theatre)** | The nurse incorporates a spiritual practice within clinical safety boundaries, strengthening trust without compromising care. | Negotiated cultural accommodation | Culture can support relationally acceptable evidence-based care | **Cultural Priorities versus Evidence-Based Care** |
| “All I got were generic guidelines to ‘keep calm.’” **(P11, Surgical Ward)** | Abstract expectations of professionalism leave the nurse without practical support for responding to patriarchal conflict. | Unsupported professionalism | Educational insufficiency individualises a systemic ethical burden | **Systemic Resource Gaps and Educational Shortfalls: A Widening Chasm Between Policy and Practice** |
| “Policy points in a direction, without helping us cross the cultural gap.” **(P13, Medical Ward)** | Policy communicates expected outcomes without supplying the training, authority, or institutional support required for implementation. | Implementation without accompaniment | The policy–practice divide constrains nursing moral agency | **Systemic Resource Gaps and Educational Shortfalls: A Widening Chasm Between Policy and Practice** |
| “Political connections or social ranking can overrule it.” **(P2, Emergency Department)** | Anticipated political repercussions narrow the nurse’s perceived capacity to advocate for clinically appropriate care. | Politically constrained advocacy | External social power displaces nursing knowledge and clinical authority | **Confronting Hierarchies in Nurse–Patient Interactions** |
| “That level of advocacy is a privilege of my seniority.” **(P6, Oncology Nursing)** | Professional experience and established institutional relationships increase the nurse’s capacity to resist family pressure. | Seniority as professional capital | Accumulated credibility can make social and professional hierarchies more negotiable | **Confronting Hierarchies in Nurse–Patient Interactions** |
