# Supplementary material for: Ethics in the crossfire: politicised patient care, cultural authority, and nurses’ constrained moral agency
Source: BMC Nurs. 2026 Aug 10;25:1080. doi: 10.1186/s12912-026-05199-2 (PMC13741607; doi:10.1186/s12912-026-05199-2)
Supplement: Supplementary file 3 [file 12912_2026_5199_MOESM2_ESM.docx]

**Supplementary Table S1. Core Interview Questions**

1. Could you describe a recent encounter where patient or family beliefs were at odds with standard clinical practice?
2. In what ways do you respond when patients’ political, cultural, or religious views appear to influence their care decisions?
3. What strategies do you employ to balance respect for patient autonomy with professional guidelines in such circumstances?
4. Have you ever experienced a situation where your personal beliefs clashed with those of patients or their families?
5. How have new laws or institutional policies, if any, affected your approach to ethically charged patient interactions?
6. Can you discuss an instance where differing viewpoints among family members, colleagues, or administrators impacted care delivery?
7. In your experience, how does communication shape the resolution of ethically complex or politically influenced cases?
8. Could you elaborate on the forms of support or guidance you receive from your workplace or professional networks when facing these dilemmas?
9. How do you interpret the term “politicized patient care,” and how does it manifest in your nursing practice?
10. Based on your experiences, what developments in nursing education or policy reforms would enhance nurses’ ability to manage ethically challenging scenarios?
